# The value of Holter monitoring in the assessment of Pediatric patients

**Published:** 2007-10-22

**Authors:** Ranya A Hegazy, Wael N Lotfy

**Affiliations:** 1 Lecturer of Pediatrics, Faculty of Medicine, Cairo University; 2 Asst. Professor of Pediatrics, Faculty of Medicine, Cairo University

**Keywords:** Holter, pediatric, arrhythmias

## Abstract

**Aims:**

Holter monitoring (HM) has been established as one of the most effective noninvasive clinical tools in the diagnosis, assessment and risk stratification of cardiac patients. However, studies in the pediatric age group are limited. The present work aims at determining the value of HM in the diagnosis and management of children.

**Settings and Design:**

Retrospective study conducted at a tertiary referral arrhythmolology service.

**Methods and Material:**

Holter records of 1319 pediatric patients (54.1% males and 45.9% females) were reviewed. Their average age was 6.7± 4.1 years (5 days-16 years). Indications for which Holter monitoring was done were analysed as well as all the abnormalities diagnosed and factors that may increase Holter yield.

**Statistical analysis used:**

Statistical Package of social science (SPSS) version 9,0 was used for analysis of data.

**Results:**

The most common indications were palpitations (19.8%), syncope (17.8%), cardiomyopathy (12.6%), chest pain (10%), evaluation of antiarrhythmic therapy (6.8%), postoperative assessment (2.6%) and complete AV Block (2.4%). A  sum of 141 Holter recordings were found abnormal with a total diagnostic yield of 10.7%. The highest contribution to diagnosis was in postoperative assessment (32.4%) and in cardiomyopathy (19.9%) where the most common abnormalities were frequent supraventricular / ventricular premature beats, supraventricular tachycardia, ventricular tachycardia and AV block. Diagnostic yield was low in  patients with palpitations (5.7%) and syncope (0.4%). An abnormal ECG was significantly associated with a higher diagnostic yield (p=0.0001). None of the children with chest pain had abnormal Holter recordings.

**Conclusions:**

HM has an extremely valuable role in the assessment of high risk patients (postoperative and cardiomyopathy). However in children with palpitations, syncope and chest pain HM has a low yield. In this group of patients an abnormal ECG is more likely to be associated with abnormal Holter recordings.

## Introduction

More than 4 decades of clinical experience have shown long-term ECG to be one of the most effective non invasive clinical tools in the diagnosis and assessment of cardiac symptoms, prognostic assessment or risk stratification of various cardiac populations and in the evaluation of many cardiac therapeutic interventions [1]. It remains an indispensable and highly refined tool for cardiac rhythm analysis and risk prediction [2].

The clinical utility of ambulatory ECG lies in its ability to examine continuously a patient over an extended period of time, permitting patient ambulatory activity and facilitating the diurnal electrocardiographic examination of a patient in a changing environmental milieu (both physical and psychological) [3].

However, the value of Holter monitoring in capturing brief symptomatic episodes of conduction system disorders is limited by the fact that they have to occur often enough to be captured during the 24 hour period of recording [4]. Despite its vast application in adult patients, studies in the paediatric age group are limited. The present work aimed to determine the value of Holter monitoring in the diagnosis and management of paediatric patients. The indications for which Holter monitoring is done for were revised.

## Subjects and Methods

### Study population

The present work reviewed the Holter monitorings of 1319 pediatric patients presenting to the Arrhythmology clinic, department of Paediatrics, Faculty of Medicine, Cairo University; a tertiary referral center for paediatric patients; in the time period between June 2000 and June 2006.

The patients were referred for the following indications:

1. Screening for arrhythmias in patients with: syncope, palpitations, chest pain, mitral valve prolapse (MVP), WPW, rheumatic heart disease (RHD) and abnormality on examination (rapid - slow - irregular heart)
2. Follow up of patients with complete AV block (CAVB), pacemaker, on antiarrhythmic therapy, frequent ventricular ectopics (VE) and postoperative assessment.
  In children with complete heart block, a decision needs to be made about their need for pacing. A lot of indications for pacing as stated by the AHA can be deduced from 24 hour HM. These include the presence of long pauses, the minimum heart rate and the presence of non benign arrhythmias.
  With our limited resources we need to prioritise patients and hence pacing is not a routine procedure for all children with complete heart block but only when indicated. 24 hour Holter monitoring helps us make such a decision.
3. Patients with cardiomyopathy (dilated, hypertrophic and restrictive)

### Methods

Ambulatory 24 hour Holter recordings were obtained in a standard fashion with 3-channel (V1, V5, aVF ) PC card recorders

#### Analysis

All Holter recorders were subsequently analysed using a vision™ analysis, Spacelab Burdick. This analysis system uses a feature extraction system to group individual QRS complexes based on their features and utilizes technician interaction in arrhythmia analysis aided by visual superimposition to correct for artefact and any erroneous analysis. In each case the data were revised and verified by both authors.

*Heart rate:* Maximum, minimum and mean heart rates over the length of the analysis were measured.

*Respiratory sinus arrhythmia* was identified if PP intervals were present that exceeded the previous PP interval by more than 10% (and not 2:1 SA block).

*1st degree AV block* was defined as PR interval of:

- More than 0.18 sec in 0 months - 6 years
- More than 0.2 sec in 6 - 12 years
- More than 0.22 sec in older than 12 years

*Supraventricular arrhythmias:* number of premature beats in 24 hours was measured by counting them during every hour of recording and computing the total.

SVT  ≥ 3 consecutive supraventricular extrasystoles.

VT    ≥ 3 consecutive ventricular extrasystoles.

*Pauses* are identified when an RR interval exceeds preset criteria. This was set at 2 sec for all patients included in the present work. Although we acknowledge the fact that ECG changes do happen in sleep, separating waking from sleeping hours in pediatric patients is not an easy job. Most parents do not fill out diaries in younger patients, and most patients have quite an irregular sleep pattern with their Holter recorders on. So we chose to define the absolute limits for pauses and bradycardia in children.

Recordings with less than 21 hours of analysable data due to noise, loss of signal or artefact were not included in the present study.

## Statistical Analysis

Statistical Package for Social Science was used for analysis of data. Data was summarized as mean, SD and percentage. Chi Square test was used for analysis of qualitative data. P-value was considered significant if <0.05.

## Results

1319 Holter records were reviewed. Patients had an average age of 6.7 ± 4.12 years with a range of 1 month - 16 years. 713 (54.1%) males and 606 (45.9%) females were included. The patients' Holter recordings were done due to the indications shown in Table 1.

A positive contribution of Holter was identified as:

1. *Diagnosis:* Establishing a new diagnosis / changing a current diagnosis  / confirming a suspected diagnosis.
2. *Management:* Introducing new therapy /withdrawal of current therapy / change of current dosage of therapy.

Holter monitorings were of positive contribution to diagnosis in 144 (10.9%) of patients and to management in 258 (19.6%) of patients. Contribution in the different patient groups is shown in  Table 2.

### Abnormalities

 141 patients (10.7%) had abnormal Holter recordings (Table 3). 299 patients (22.6%) had infrequent isolated supraventricular  ectopics and 1185 patients (89.9%) had infrequent isolated ventricular ectopics. These were not considered abnormal. Arrhythmias in the different patient groups is shown in Table 4. 12 lead ECG was performed in 1136 (86.1%) of patients prior to Holter recordings. Of those 298 (26.2%) had abnormal ECGs. 77 (49.4%) of patients with abnormal ECGs had abnormal Holter recordings as compared to 64 (5.6%) of patients with normal ECGs (p= 0.0001). The presence of an abnormal ECG was significantly correlated to the contribution of Holter recording as regards diagnosis (p=0.0001) and management (p=0.0001) (Table 5).

9.6% of patients were symptomatic during Holter recordings, however abnormalities were detected more frequently in asymptomatic patients. Only 168 (12.7%) of our patients took a patient diary despite the fact that the importance of the diary was explained to all patients. This may be related to illiteracy, lack of compliance and inability of parents to correlate to their children"s symptoms especially in the younger age group. 25 of these had an abnormal Holter recording. Although patient diary didn't increase the contribution of Holter monitoring to diagnosis (p=0.3), or management (p=0.2). Yet in those who took a diary a follow-up Holter was needed in a significantly less number of patients (p=0.02). This is explained by the fact that when a diary was taken we could more confidently exclude arrhythmias as a cause for the patient's symptoms and hence no follow up HM would be recommended.

## Discussion

Although widely used in arrhythmic disorders of adult patients, studies in the paediatric age group are limited [5]. Holter technology has endured for more than 40 years and proven to be a valuable adjunctive non-invasive diagnostic technology to record the ambulatory long term ECG [1].

Children are referred for 24 hour ECG monitoring for many indications [6]. The present work, which included 1319 paediatric patients, showed that the commonest indication, for which children were referred, was for the evaluation of palpitations. 8.8% of the examined recordings revealed abnormalities, out of which n=15 (5.7%) of diagnoses were established solely on   and n=17 (6.5%) had a change of their management. These patients had a higher percentage of pauses, SVE, VE and HB than those who presented with other symptoms which is in accordance with Ayabakan et al, 2000. We thus support the recommendation that one of the primary indications of AECG monitoring is to exclude arrhythmias as the cause of palpitation [6].

Cardiac arrhythmias should be considered among the malignant causes of syncope in children 7,8. Hence syncope is a common cause of referral for Holter (17.8% n=234 patients). Although abnormalities were detected in 14 (5.9%) of patients, only one patient with sick sinus syndrome had his diagnosis solely established according to Holter monitoring. The low diagnostic yield of Holter in children with syncope had been previously reported [5,9,10]. On the other hand Kilic et al., [7] reported a much higher diagnostic yield of AECG, but the highly selective nature of the study population most likely contributed to this fact. An earlier study by Drago et al. [11], also concluded the diagnostic usefulness of Holter in paediatric fainting reporting a high diagnostic yield compared to resting ECG.

In the present study, 20 patients reported syncope on the day of the recording despite maintaining sinus rhythm. In these patients AECG was valuable in excluding arrhythmias as a cause for syncope. Still a comprehensive work up of paediatric patients with syncope that includes careful history and ECG is essential prior to Holter monitoring to increase yield [8,9,12,13].

AECG provides useful prognostic information in patients with DCM and may identify several independent predictors of mortality including nonsustained VT and mean HR [14]. The present work included 166 cardiomyopathy patients, with a diagnostic yield of 20% of patients and an even higher percentage of 36.1% had a change of management. According to our work we believe that AECG remains an indispensable tool in the regular assessment and risk stratification of children with cardiomyopathy with important implications on their diagnosis and management.

Chest pain is a common cause of anxiety among patients and parents and it is one of the most frequent causes of referral to cardiology outpatient clinics [15,16]. Despite that, chest pain in children is very rarely caused by a significant cardiac pathology [16-18]. None of our patients who presented with chest pain had abnormal Holter recordings. Hence there seems to be a general agreement that in chest pain particularly in the absence of organic heart disease; Holter monitoring has little value [6,19]. The primary role of HM in this case may be to exclude rather than diagnose a cause.

HM has been documented to provide insight in patients referred for arrhythmias suspected on physical examination [20]. A diagnostic yield of 20.1% in patients who had HM done based on abnormality detected on examination (rapid heart / slow heart/ irregular heart) and another 17.6% had a change of management. Hence it should be considered as an indication for HM. However, the present work didn't differentiate those referred from general practitioners and those from specialized cardiologists to evaluate difference in yield.

In some studies, HM led to predictions of antiarrhythmic drug efficacy more often than did electrophysiological testing and there was no significant difference in the success of drug therapy as selected by the two methods, hence favouring the non-invasive former method [21]. Follow up of antiarrhythmic drug efficacy during periods of somatic growth is a class I indication for HM [19]. We found that Holter monitoring is an excellent method for assessment of antiarrhythmic drug efficacy where 72.2% of patients had a change of their management based on their HM. We agree with the fact that this number would seem optimistic, however we are merely reporting our experience where we found that HM was sensitive enough to detect the occurrence of arrhythmias in the studied children kept on antiarrhythmic therapy. The change of management referred to, was mostly in the form of changing the dosage of a current therapy, adding a new drug or withholding treatment. This was done in correlation with patient symptomatology and accurate history taking.

In a study by Jurkovicova et al. [22], HM was found to be an unsuitable method for estimation of antiarrhythmic therapy of PSVT because of the considerable spontaneous variety. This was generally not reported by others.

Diagnostic yield was 29.4% in patients with prior surgical treatment of their congenital heart disease and 26.5% had a change in their management based on their HM. However the present work didn't specify which cardiac defect surgeries were associated with higher yields. This is important to consider, as using AECG in periodic evaluation of these patients must be based on consideration of the type of defect, ventricular function and risk of late postoperative arrhythmias [23]. However, surgical treatment of Fallot tetralogy is one of the most common cardiac procedures at our institution and previous reports have established Holter monitoring as a non-invasive and very sensitive method for discovering heart rhythm disturbances in children after the repair of Fallot's tetralogy [24,25].

HM has been found extremely valuable in determining predictive factors for Adams stokes attacks and heart failure in children and adolescents with complete heart block [26]. Non-paced children need regular Holter monitoring assessments to complete their diagnostic work up and help decision making [19]. We found Holter monitoring a corner stone in decision making of children with CHB. We also found HM a useful method in determining the clinical characteristics and further management in patients (52.2%) with frequent VE on their ECGs with a diagnostic yield of 39.1%. We agree with Tsuji et al. [27], that HM is valuable method in the long term follow up of these patients.

There is evidence that the incidence of potentially lethal ventricular arrhythmias in patients with MVP particularly with significant MV regurgitation is greater (but only marginally) than the general population [28-30]. However our study showed a low diagnostic yield in children with MVP and the value of AECG in routine evaluation of these patients remains controversial. We however included all patients with MVP regardless of the degree of their mitral valve regurgitation which may have influenced our yield. Nevertheless guidelines regarding Holter monitoring in MVP need clarification.

Patients, who are paced due to CHB, whether congenital or acquired, have regular HM at our arrhythmology service. The present work showed that 82% of paced patients had changes in their management based on their HM. Change of management was in adjusting pacing settings and in 2 patients loss of capture was diagnosed and they had to have a new pacemaker inserted.

The high diagnostic yield associated with long term ECG is not simply related to the prolonged time of examination, but has been facilitated by Holter technology, which permits the detection, recognition, amplification and recording of the pacing stimulus artefact [3].

Although diagnostic assessment and treatment have been described in detail in patients with symptomatic WPW the management of asymptomatic subjects remains controversial [31]. Holter monitoring was deemed unnecessary in evaluation of asymptomatic patients with WPW [19]. Usually these patients are assumed to have a benign process, however very occasionally they present with VF as the first manifestation of the syndrome [31] hence the dilemma of how extensively they should be investigated and managed. Despite the fact that it has been concluded that patients with WPW who develop AF and VF are different from those who don't [32]; non-invasive methods as HM seem to be relatively incomplete for risk stratification [31]. Our experience included only 4 patients with asymptomatic WPW of which one patient had abnormalities. This number is statistically too small to deduce conclusions or recommendations.

Data are limited regarding prevalence of arrhythmia in RHD patients and Holter monitoring is not part of their routine diagnostic work up. The low diagnostic yield in RHD in the present work is supportive of this notion.

Although taking a diary was not associated with increase in diagnostic yield or change of management among the patients included in the present study, follow up was significantly needed in less patients; saving time and effort. This emphasizes the need to stress on taking a diary. This will at least exclude arrhythmias as a cause of symptoms if not establish it. When patients or parents forget to document the timing of the symptoms, the specificity of subsequent interpretation is reduced [33].

An abnormal ECG is a clue to increase yield of subsequent Holter monitoring. This is likely due to the fact that patients with structurally abnormal hearts or with cardiac rhythm abnormalities evident on their resting ECG are more likely to have abnormal Holter monitoring [34].

The heart rates of children included in the present work after excluding those with structurally abnormal hearts are presented in an age stratified manner. This sample of children is larger than those previously reported [34], and can be used as a basis for heart rates among ambulatory healthy children. It's not uncommon to hear colleagues opine that HM is outdated especially with the advent of more sophisticated monitoring techniques. However, HM has proven to be complementary or even superceding other technologies as event recorders and TTM [35,36].

## Conclusion

HM has an extremely valuable role in the assessment of high risk patients (postoperative and cardiomyopathy). However in children with palpitations, syncope and chest pain HM has a low yield. In this group of patients an abnormal ECG is more likely to be associated with abnormal Holter recordings. Holter monitoring generally satisfies the physician's demands if expectations do not exceed the limitations of the method.

## Figures and Tables

**Table 1:**
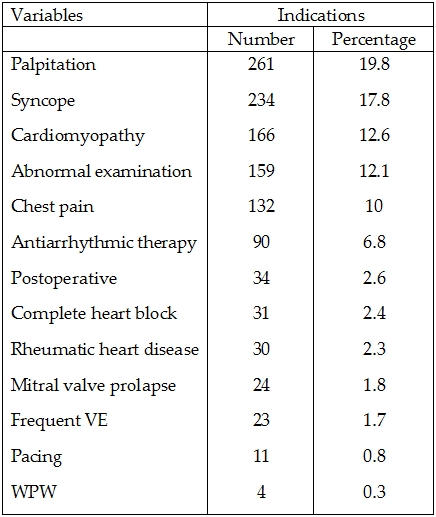
Indications of Holter recordings in patients included in the study

**Table 2:**
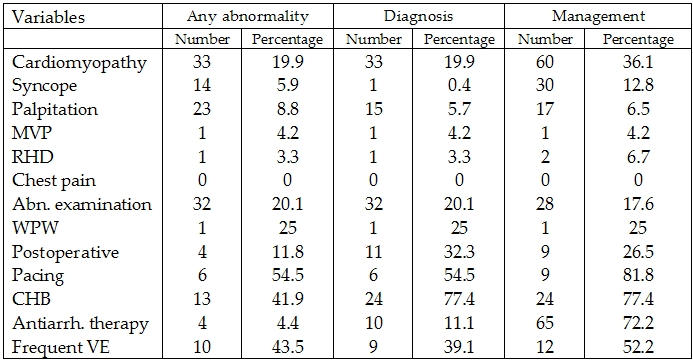
Positive abnormality, diagnosis and  management of patients included in the study

**Table 3:**
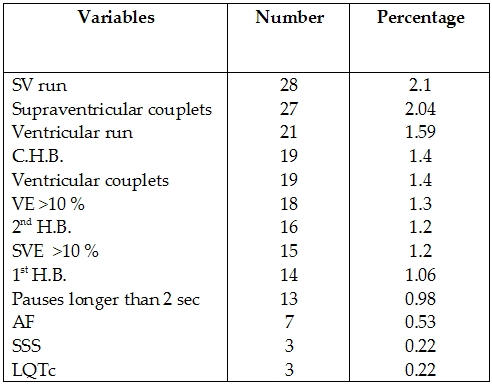
Different type of abnormalities diagnosed by Holter for patients included in the study

**Table 4:**
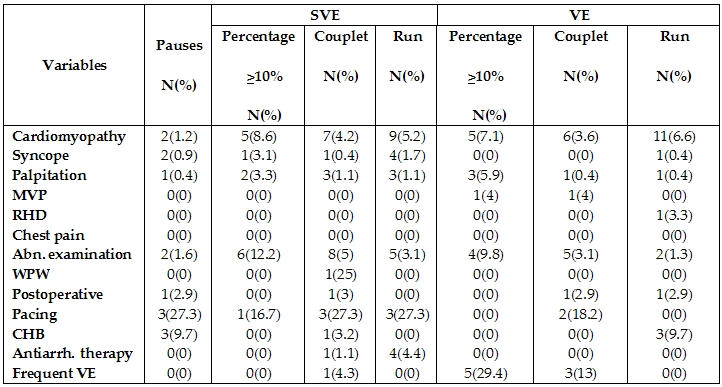
Pauses, SVE and VE  of patients included in the study in different indications

**Table 5:**
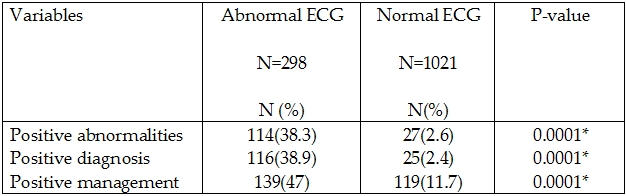
ECG findings in relation to abnormalities, diagnosis and management of patients included in the study

* P-value is significant if <0.05
